# Inadequate linear catch-up growth in children born small for gestational age: Influencing factors and underlying mechanisms

**DOI:** 10.1007/s11154-024-09885-x

**Published:** 2024-05-20

**Authors:** Anran Tian, Fucheng Meng, Sujuan Li, Yichi Wu, Cai Zhang, Xiaoping Luo

**Affiliations:** Department of Pediatrics, Tongji Hospital, Tongji Medical College, Huazhong University of Science and Technology, Wuhan, 430030 China

**Keywords:** Small for gestational age, Catch-up growth, Growth hormone-insulin-like growth factor axis, Growth plate, Short stature

## Abstract

A minority of children born small for gestational age (SGA) may experience catch-up growth failure and remain short in adulthood. However, the underlying causes and mechanisms of this phenomenon are not yet fully comprehended. We reviewed the present state of research concerning the growth hormone-insulin-like growth factor axis and growth plate in SGA children who fail to achieve catch-up growth. Additionally, we explored the factors influencing catch-up growth in SGA children and potential molecular mechanisms involved. Furthermore, we considered the potential benefits of supplementary nutrition, specific dietary patterns, probiotics and drug therapy in facilitating catch-up growth.

## Introduction

Small for gestational age (SGA) is generally defined as the birth weight and/or birth length of infants that is less than 2 standard deviation scores (SDS) from the mean for gestational age [1]. Based on the reference data, neonates can be subdivided into SGA for weight, SGA for length, or SGA for both weight and length, which helps to understand the mechanisms and effects of being bore SGA [1]. Noteworthy, birth weight is currently the most often used reference data to define SGA newborns.

Catch-up growth in linear growth refers to “a height velocity above the statistical limits of normality for age and(or) maturity during a defined period, following a transient period of growth inhibition” [2]. The typical growth pattern of SGA children is defined as a period of accelerated linear growth, which occurs mainly in the first 12 months of life, with a complete recovery in height at the age of 2 years [3]. However, a landmark study by Karlberg et al. in 1995 reported that 10–15% of full-term SGA children lack catch-up growth, and most of these children remain short in adulthood [4]. For late preterm SGA children, one-third of them were below the 10th percentile for length at 36 months of corrected age [5]. Currently, the treatment of recombinant human growth hormone (rhGH) has been approved by many countries for short children born SGA. However, the treatment is less effective than in patients with growth hormone deficiency (GHD) [6]. In addition, the timing of initiating growth hormone therapy is controversial due to the uncertainty of spontaneous catch-up growth in children with SGA [7]. This variability may be attributable to multiple genetic variations and other causes of perturbation of linear growth [7, 8]. Therefore, exploring the mechanisms affecting insufficient linear catch-up growth is necessary for optimizing the adult height of short SGA children. Although several studies on SGA children describe catch-up growth in both height and weight, we focus on catch-up growth in height in this review.

Here, we review the role of the growth hormone-insulin-like growth factor axis and the growth plate in SGA children lacking catch-up growth, the numerous factors affecting catch-up growth, and the possible molecular mechanisms. Lastly, we conclude with a discussion of potential therapeutic approaches for short SGA children.

## Main text

### Etiology of SGA

Maternal, placental, and fetal factors interact in a complicated way to cause the multifactorial etiology of SGA [1, 9]. Maternal nutrition, health status, medications, habits, and genetic factors (e.g., height, weight, and uterine capacity) contribute to the growth of the fetus. Placental insufficiency and anomalies prevent the placenta from providing an appropriate quantity of nutrition and oxygen to the fetus, which most frequently occurs in pre-eclampsia. Fetal factors such as multiple genetic syndromes and perinatal infections may affect offspring birth size.

The etiology of SGA may affect catch-up growth. Fang et al. classified the probable causes of SGA into five etiologic subgroups, and investigated the effect of each group on the risk of growth restriction in children with SGA at age 7 years for the first time [10]. This study concluded that low maternal height was a physiological factor, while smoking during pregnancy was an environmental factor. SGA patients with mothers whose heights were less than the third percentile had the highest risk of short stature at age 7 years (the adjusted odds ratio (aOR) = 6.04, 95% CI 3.93–9.27). The fetal etiology group came in second, with emphasis on growth limitation at age 7 years due to serious birth defects such as congenital heart disease, maternal viral infections during pregnancy, Down syndrome, and major birth defects that may influence fetal growth and development. (aOR = 4.78, 95% CI 3.41–6.71) [10]. SGA children with unknown risk factors, maternal factors, placental factors, and environmental factors also had a higher risk of growth restriction at 7 years. (aOR = 3.84, 95% CI 2.64–5.58, aOR = 3.47, 95% CI 2.62–4.60, aOR = 3.36, 95% CI 2.51–4.46, aOR = 2.19, 95% CI 1.59–3.03, respectively) [10]. Unknown risk factors may indicate undiagnosed genetic defects. It is important to note that the placental etiology subgroup may be at higher risk for growth restriction than the study reflects because the most important placental factor, pre-eclampsia, was considered a maternal factor in this study.

Overall, all infants with maternal, fetal, environmental, placental, and genetic variables that contributed to SGA had a higher chance of growth limitation at age 7 years than normal appropriate for gestational age (AGA) infants. Our review will discuss and explore their respective characteristics and possible mechanisms of catch-up growth below.

### Pathophysiology in short SGA children

There have been two main accepted models of catch-up growth. One is the neuroendocrine hypothesis, which was proposed by Tanner in 1960s [11]. It suggested that the hypothalamus can compare body size with the ideal size of an individual at that age (possibly encoded by a gene). When there is a mismatch, the body grows faster than average by adjusting growth factors. The other one is the growth plate hypothesis. Animal studies suggested that growth plate chondrocytes have a limited ability to proliferate. After a brief period of growth inhibition, the growth plate showed remarkable ability to proliferate; therefore, catch-up growth occurs [12]. Since then, many hypotheses have been extended from these two hypotheses [13]. Roselló-Díez et al. provided an extensive discussion on catch-up growth in 2015 [2]. It clarified that catch-up growth was a combination of the two primary recognized concepts and occurred at three levels: the cell itself, the tissue, and the collaboration between different organs. Cells have their own "growth program", after which all cells within a tissue coordinate with each other through a common program. Ultimately, the coordinated growth between different organs relies on intrinsic programs and extrinsic hormones.

#### The GH/IGF-1 axis

GH/IGF-1 axis plays an extremely significant role in longitudinal growth and development [14]. The function of growth hormone (GH) depends on the growth hormone receptor (GHR). GHR affects insulin-like growth factor 1 (IGF-1) gene expression and thus leads to IGF-1 secretion [15]. Most circulating IGF-1 is produced by the liver and is regulated prenatally by insulin and postnatally by GH. Circulating IGF-1 binds mainly to IGF-binding protein 3 (IGFBP-3) and IGF-binding protein 5 (IGFBP-5), which in turn form a ternary complex with acid-labile subunit (ALS), significantly prolonging the half-life of the circulating IGF1-IGFBP binary complex [16]. IGF-1 acts by binding to IGF-1 receptors (IGF1R). IGF1R binds to the ligand, leading to autophosphorylation and tyrosine phosphorylation. Subsequently, substrate phosphorylation activates two major signaling pathways, the phosphatidylinositol 3-kinase (PI3K)/ protein kinase B (AKT)/mammalian target of rapamycin (mTOR) and RAS/mitogen-activated protein kinase (MAPK) [17]. After that, GH-IGF-mTOR/MAPK pathway can be activated by multiple factors such as nutrition [18]. Activation of the GH-IGF-mTOR/MAPK pathway initiates numerous fundamental cellular processes in organ and body growth and development [19, 20]. In growth plates, GH-IGF-mTOR pathway is responsible for the linear growth of the long bones [19].

**Fig. 1:**
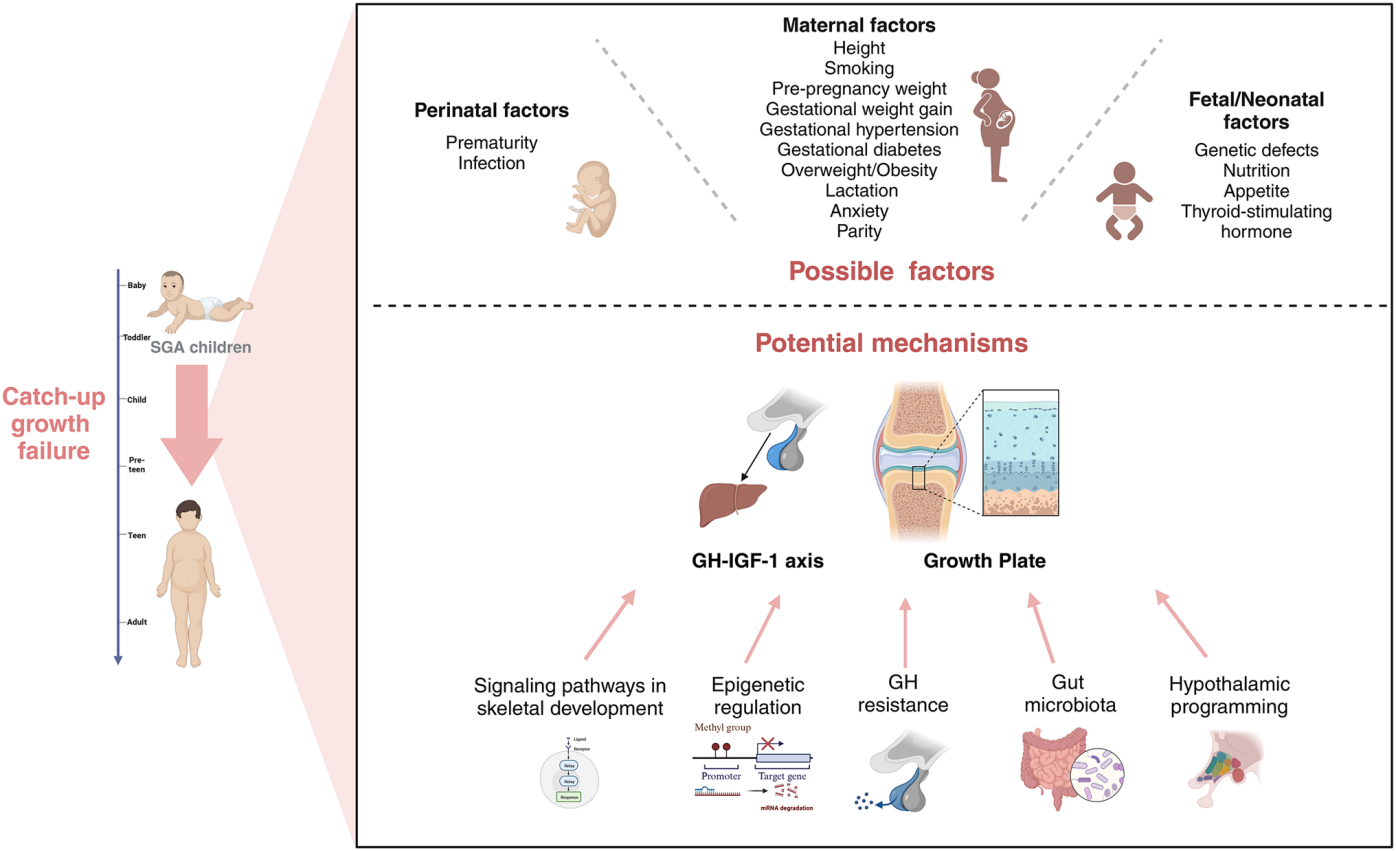
The possible factors and potential mechanisms of catch-up failure in SGA children. Disorders of the GH-IGF-1 axis and abnormal cartilage ossification within the growth plate are the main pathological features of short SGA children. Multiple maternal, perinatal, neonatal and fetal factors impaired the ability of SGA infants to catch up. Signaling pathways in skeletal development, epigenetic regulation, GH resistance, gut microbiota, and the reprogramming of the hypothalamic–pituitary–adrenal axis are the possible mechanisms for catch-up growth failure

In the intrauterine period, fetal growth retardation is manifested by reduced insulin and IGF-1 levels [21]. After birth, it was reported that the SGA neonates showed resistance to GH and IGF-1 compared to those AGA infants [22]. Then growing up for a while, the short SGA children also rarely developed typical GH deficiency, but there were abnormalities in the GH/ IGF axis [23]. (Fig. 1).

Several studies revealed that, as compared to controls, persistently short SGA children exhibited lower spontaneous GH secretion [24]. In particular, short SGA children under 6 years of age have alternative GH secretion patterns including high basal GH levels, high peak frequencies, but low peak amplitudes [24]. This aberrant GH pattern, however, may be a phenomenon specific to a certain age range, as it ceases in children with SGA beyond the age of 6 years [25]. Overall, the current study shows that low spontaneous GH levels, rather than altered secretory rhythms, remain the primary cause of aberrant GH secretion in short SGA and this difference in secretion rates is age-related, with GH secretion rates increasing with age.

Due to lower natural GH secretion, the IGF-1 levels were much lower in the non-catch-up growth subgroup compared to the SGA children who caught up in growth [26]. This supports the hypothesis that IGF-1 and childhood height are positively associated [27]. Additionally, in children treated with rhGH, it was found that short children with SGA may have some degree of IGF-1 resistance because they required higher GH-induced IGF-I levels to achieve growth rates similar to those of children with familial short stature or GHD [22]. SGA children with this mildly aberrant IGF-1 signaling pathway are common; about half of the short children born SGA fulfill the criteria for IGF-1 resistance [28]. Indeed, the unique physiological circumstances and genetic heritage of short SGA children may be the root of the debate regarding IGF-1 levels. If SGA children showed normal GH secretion with IGF-1 resistance, they often have mutations in either the *IGF1R* gene or the *IGF1* gene [28]. When the baseline GH level is naturally low, factors influencing IGF-1 sensitivity could be identified, such as administration of exogenous rhGH, dosage adjustments for weight gain, treatment compliance, and characteristics of the study population (including younger age, delayed bone age, extreme shortness, and partial syndromes) [29].

#### The growth plate

Endochondral ossification is the pathway for organogenesis and skeletal elongation. The rate of postnatal bone growth is almost entirely dependent on the cartilage growth plate [30]. The growth plate is located between the epiphysis and the metaphysis of long bones and is divided into three well-defined zones. The resting zone (RZ) closest to the epiphysis generally serves to support the proliferative zone (PZ). PZ, directly below the RZ, begins to divide and form a columnar arrangement. Then, in the hypertrophic zone (HZ), the chondrocytes initiate terminal differentiation [31].

Long bones lengthen with the proliferation and hypertrophy of growth plate chondrocytes and are closely controlled by systemic endocrine molecules. The major endocrine factors regulating the growth plate include GH, IGF-1, thyroid hormones, and so on. Indian hedgehog (IHH), parathyroid hormone-related peptide (PTHrP), and fibroblast growth factors (FGFs) are examples of autocrine/paracrine molecules that act locally in the growth plate [18, 32]. They are all responsible for helping the growth plate transition from the proliferative zone to the hypertrophic zone and function correctly [32]. The rabbit model demonstrated that during catch-up growth, the proliferative zone, hypertrophic zone, and total growth plate all experienced a delayed decline in senescence. This suggests that, at least in part, the delay in growth plate senescence is what allows for greater proliferative capacity during linear catch-up growth. By modifying the cellular senescence process, it might be feasible to encourage catch-up growth in SGA children more successfully [33].

Recent studies suggested that SGA children with inadequate catch-up growth had abnormal skeletal development. SGA infants had significantly lower femur, tibia, humerus lengths and cortical bone mass than AGA infants [34]. When SGA children experience catch-up growth in height postnatally, they can reach the average long bone length and mineralization by 4 years of age [35, 36]. Still, SGA children who lack catch-up growth have insufficient bone mineral accumulation during growth and are at risk for low adult bone mass [37]. Cohort studies highlight the bone mineralization is the outcome of catch-up growth. Hormone level improvements alone, such as exogenous rhGH injection, are not sufficient to promote bone formation in short SGA cases. Research by Schweizer et al. revealed that rhGH treatment did not increase bone diameter in children with short SGA [38, 39]. The above-mentioned studies showed that regulatory problems at two levels—growth plate senescence control and endocrine system hormone regulation—are the cause of catch-up failure in SGA children. The limitation of the study is that the subjects were only full-term SGA children. In children with preterm SGA, a severe prenatal shock may have a direct impact not only on growth catch-up but also on bone formation.

### Multiple influencing factors in inadequate catch-up growth in SGA children

#### Maternal factors

Maternal factors not only cause neonates to exhibit SGA at birth, but further affect postnatal catch-up growth in SGA children. These determinants cover not only maternal physiological characteristics (e.g., height, weight, weight gain during pregnancy, placental function, and illnesses during pregnancy), but also maternal psychological states (e.g., anxiety, depression) and breastfeeding behaviors. A study analyzing a subgroup of the Early Childhood Longitudinal Study Birth Cohort (ECLS-B) found that the effects of short maternal height, pre-pregnancy underweight, inadequate gestational weight gain, and smoking on poor catch-up growth before school age [7]. Placental insufficiency alters postnatal growth trajectories in very low birth weight (VLBW) children, but early placental insufficiency does not appear to affect height at 12.5 years of age in children with SGA [40, 41]. More research is therefore needed to focus on the impact of varying degrees and timing of placental insufficiency on the early growth patterns of children with SGA. Another prospective cohort study also showed that gestational hypertension, gestational diabetes, and maternal overweight/obesity were inversely associated with catch-up growth in SGA children [42]. Preeclampsia, a hypertensive disorder of pregnancy, may include pathologically varied disorders that are categorized according to severity or onset [43]. The effect of preeclampsia on offspring’s longitudinal growth is heterogeneous, and there have been no investigations evaluating preeclampsia as an independent factor for catch-up growth in the SGA state [43].

Moreover, some maternal factors universally affecting postpartum growth in offspring could also have a great impact on the catch-up growth of SGA children. These factors are often associated with abnormalities in maternal lactation. An analysis of the Avon Parent–Child Longitudinal Study (ALSPAC) cohort in the United Kingdom showed that the degree of maternal anxiety was negatively associated with children's BMI in the second year of a child's life [44]. Recent researches supposed maternal depression during pregnancy causes delays in breastfeeding initiation and affects breastfeeding patterns [45, 46]. Furthermore, parity played a role in growth, as another analysis of the ALSPAC cohort showed that infants of first-time mothers go through rapid catch-up growth in weight and length during the first year of life, with significant increases in weight and length from 12 months of age [47]. Parity's effect on the timing of the initial breastfeeding might be the reason for inadequate catch-up growth in SGA children [48].

#### Perinatal factors

Preterm birth is another factor of insufficient catch-up growth. A community-based cohort study showed that children born preterm SGA had an increased likelihood of postnatal growth restriction compared to full-term SGA children and preterm AGA children. About 30–39% of preterm SGA children aged four could not catch up on growth, compared with only 5% of preterm AGA children and 9% of term SGA children who do not catch up [49]. The study revealed that premature birth and SGA status jointly affect growth potential, which was likely due to the neonatal complications and chronic diseases related with preterm birth, affecting early growth [50, 51].

Perinatal infection also causes catch-up growth failure in SGA children. Congenital infections caused by *Toxoplasma gondii*, rubella, cytomegalovirus (CMV), herpes simplex virus (HSV), varicella-zoster virus (VZV), and *Treponemes* not only cause SGA in newborns, but may also further affect its postpartum growth potential [52]. An observational study found that newborns with SGA with CMV infection have slightly different growth patterns than those with general SGA. Typically, 90% of SGA tend to experience accelerated catch-up growth in the first two years. However, the catch-up rate among SGA children with CMV infection is about 75% [53]. Multiple causes contribute to the failure of catch-up growth in SGA children with CMV infection. Children with severe neurologic disease caused by CMV are often associated with nutritional difficulties and endocrine disorders, such as abnormalities in GH secretion. Second, poor motor function due to CMV may affect bone growth in children with SGA. In addition, CMV may play a direct role as a pathogen in catch-up growth failure [53].

#### Fetal/neonatal factors

Molecular genetic analyses revealed that many short SGA children exhibit genetic defects. The international consensus guide on SGA children, published in 2023, is an important compilation of the genetic reasons for SGA children's short stature [1]. A genetic analysis of 176 SGA children with persistent short stature showed that 42% of them had pathogenic or potentially pathogenic genetic variants (P/LP) [54]. Of all the variants detected, the most significant number of variants were those related to the growth plate, accounting for 42% of the total, with 7/74 children having variants in the short stature homeobox (*SHOX*) gene, 7/74 having variants in paracrine signaling of the growth plate, and 17/74 having variants in genes related to the cartilaginous extracellular matrix of the growth plate. 16% of P/LP gene variants affect the pituitary, GH-IGF-1, and IGF-2 axes. 3% of the P/LP gene variants affected the thyroid axis, as evidenced by *TRHR* and *THRA* variants. In 12/74 children (16%), the study also identified P/LP variants in genes involved in fundamental intracellular and intranuclear processes. The diagnosis of Silver-Russell syndrome (11p15, UPD7) was seen in 12/74 (16%) of cases, and other chromosomal aberrations were seen in 5/74 (7%) of children [54]. Genetic variations typically affect three key aspects of growth regulation: the endocrine system, growth plate function, intracellular regulation, and signal transduction processes. These align with the three regulatory levels described by the catch-up growth theory. However, the next-generation sequencing (NGS) used in research cannot identify epigenetic alterations. Hence, toxicant-induced epigenetic changes caused by maternal factors, such as smoking, alcohol consumption, and infectious diseases during pregnancy, cannot be detected.

As far as postnatal factors are concerned, nutrition and appetite play an extremely important role. Epidemiological evidence suggested that malnourished SGA children were more likely to exhibit failure to catch up on growth. Catch-up growth failure is primarily caused by malnutrition due to inappropriate breastfeeding, lack of complementary foods, infections, and other environmental factors [55]. Moreover, appetite could also be the reason for insufficient catch-up growth. Gastrin and leptin are crucial hormones that regulate appetite, food intake, and energy metabolism. Previous studies revealed that leptin and gastrin levels in the umbilical cord have predictive value for postnatal catch-up growth [56, 57]. These findings supported the possibility that satiety levels preset by the uterus may partially mediate changes in infant growth rate [58].

Average somatic growth requires that the thyroid hormone axis and the GH axis work together, and abnormalities in the thyroid hormone axis affect the GH-IGF-1 axis [59]. Postnatal thyroid stimulating hormone (TSH) in SGA children was negatively associated with postnatal catch-up growth [60, 61].

Among all the factors discussed above, genetic and epigenetic factors play major roles in postnatal catch-up growth failure, as evidenced by the fact that some SGA children have monogenic disorders, genetic defects, or epigenetic disorders such as Silver-Russell syndrome [1]. Secondly, maternal height is undoubtedly a crucial factor, which represents the limited genetic potential for linear growth in newborns and can affect the catch-up growth ability of SGA children. However, the heritability of height velocity throughout infancy is not fully understood [62]. Furthermore, in low- and middle-income nations, population height variations are more likely to be attributable to environmental factors than to genetics. Nutrition and disease are the main environmental factors affecting height [63]. For linear growth in children, intrauterine development and exposure before the age of two years are the main drivers. Therefore, physiologic conditions during pregnancy and health management during the neonatal period are particularly important for catch-up growth in children with SGA [64]. After emphasizing the magnitude of each factor, we stress that the number of SGA risk factors has a multiplier effect on children's postnatal growth. Xie et al. found that experiencing "multiple hits" in utero resulted in more severe outcomes. For example, SGA newborns with both maternal smoking and inadequate gestational weight gain during pregnancy have a much higher risk of stunting at the age 5 [7, 10].

### Potential molecular mechanisms

#### Signaling pathways in skeletal development

Genetic investigations of children born SGA with persistent short stature revealed that the majority of children in this group had genetic variations linked to growth plate development [65]. Hence, correct growth plate function is essential for SGA children to catch up on growth.

The process of forming growth plates is dynamic and strictly controlled. Numerous signaling pathways interact and are crucial to this process. The primary signaling pathways are Transforming Growth Factor (TGF)/Bone Morphogenetic Protein (BMP), FGF, WNT/β-catenin, Hedgehog, PTHrP, and notch signaling [66]. The signaling cascades converge on chondrocyte transcription factors. Runx2, a member of the Runt family of structural domains; Osterix, a member of the SP/KLF family; and Sox9, a member of the Sox family of transcription factors, all play crucial roles as transcription factors in cartilage production [66, 67]. Additionally, a number of transcriptional cofactors, including β-connexin, CBF, JAB1, and YAP1, have been demonstrated to be essential for the development of growth plates [66]. SGA children with catch-up failure exhibit not only P/LPs implicated in FGF and IHH signaling pathways, but also P/LPs influencing basic intracellular/intranuclear processes (*CDC42, KMT2D, LMNA, NSD1, PTPN11, SRCAP, SON, SOS1, SOX9. TLK2*) [54, 65]. Unfortunately, no comprehensive investigations of skeletal development in children with dwarf SGA have been conducted. CXXC finger protein 5 (CXXC5), a negative feedback regulator of WNT/β-catenin, has been shown to mediate growth plate senescence in a mouse model and is a potential target for enhancing longitudinal bone growth, offering new possibilities for diagnosis and treatment [68]. However, the expression of CXXC5 in the growth plate only increases progressively during late puberty, and its role in catch-up growth, which occurs mainly in the first two years of life, remains unclear [68]. More research is needed into the involvement of intracellular/intranuclear signaling in growth plate signaling pathways, as well as the mechanisms involved in catch-up growth failures.

#### GH resistance

The phenomenon of GH resistance has been described in short children with SGA undergoing rhGH treatment, evidenced by poorer improvements in bone strength and muscular mass compared to those with GHD [38]. Furthermore, a cohort of SGA children treated with rhGH showed varied improvements in motor function, further reflecting heterogeneity in GH sensitivity [69]. The heterogeneity in dwarf SGA patients may stem from a long-term nutritional imbalance [70]. For example, a lack of protein or other dietary components during the first two years of life can cause GH resistance [70]. In addition, lower insulin and leptin levels due to nutritional deprivation may partially mediate GH resistance by downregulating GH receptors in the liver [70]. The compensation growth pattern can explain the catch-up growth failure. GH and ghrelin levels were elevated due to low cellular nutrient levels, while IGFs and leptin were suppressed. The adverse environment increased nicotinamide adenine dinucleotide (NAD +) and adenosine monophosphate (AMP), which in turn stimulated the production of NAD-dependent deacetylase sirtuin-1 (SIRT1) and fibroblast growth factor 21 (FGF21). SIRT1 and FGF21 blocked the Janus-activated kinase 2 (JAK2) and signal transducer and activator of the transcription 5 (STAT5) signaling pathway, ultimately resulting in hepatic GH resistance [71–74]. Animal models suggested that key regulators in cytokine signaling pathways may alter downstream signaling pathways of GH, independent of postnatal nutrition. A correlation between the absence of catch-up growth and hepatic GH resistance was also observed in the uterine artery ligation-induced intrauterine growth retardation (IUGR) rat model. In addition to impaired GH-mediated JAK2/STAT5 signaling, IUGR rats without catch-up growth were found to have up-regulation of GH-induced suppressor of cytokine signaling (SOCS/CIS proteins) [75]. Therefore, in addition to postnatal nutritional deprivation, a poor intrauterine environment could lead to GH resistance by modulating SOCS/CIS expression, which further impair the offspring's catch-up growth.

#### Epigenetic regulation

Epigenetic regulation may modulate the somatotropic axis in short SGA children and result in catch-up growth failure. Pregnant women who smoke, drink alcohol, and have infectious diseases during pregnancy may result in toxicant-induced epigenetic alterations in the fetus [7]. Epigenetics refers to changes in gene expression levels due to non-genetic sequence alterations, primarily including regulation of DNA methylation, chemical modification of histones, and non-coding RNA expression [76]. Genomic imprinting is one of the most important and well-studied forms of the epigenetic phenomenon in short SGA children, which causes genes to be expressed in a parent-of-origin-specific manner. Silver-Russell syndrome (SRS), mainly driven by IGF2 / H19 imprinting domain hypomethylation in the 11p15 region, is characterized by IUGR and short stature due to catch-up growth failure [77]. However, 11p15 epigenetic mutations are rare in SGA children without Silver-Russell syndrome [78].

Methylation disorders in children born with SGA may present not only in imprinted loci but also in non-imprinted genes [9, 79]. A key characteristic of genes regulated by epigenetic modifications is their involvement in growth and development. The majority of SGA children with aberrant methylation fail to catch up on growth [79]. Animal experiments showed that prenatal exposure to inflammation leads to hypomethylation of *Mecp2 and LINE1* in the mouse hypothalamus, which may further affect the neuroendocrine system and growth potential [80].

MicroRNAs (miRNAs) may also contribute to SGA children's insufficient catch-up growth. Jeong et al. used NGS to analyze serum exosome miRNAs from 16 SGA and 10 AGA children. They found that four upregulated miRNAs: miR-30c-5p, miR-363-3p, miR-29a-3p, miR-29c-3p, and two downregulated miRNAs: miR-629-5p and miR-23a-5p were involved in SGA children without catch-up growth, and all these miRNAs were associated with cell proliferation, cell growth, IGF-1R regulation or aging process [81–83]. Mas-Parés et al. showed umbilical cord miRNAs could be novel biomarkers for the early identification of catch-up growth in SGA infants: miR-501-3p, miR-576-5p, miR-770-5p, and miR-876-3p may contribute to the regulation of postnatal height growth [84].

#### Gut microbiota

In a study of Hispanic infants in Southern California, Alderete et al. found that a more mature gut microbiota at one month of age, characterized by increased alpha diversity, predicted catch-up growth [85]. Studies on preterm infants also indicated that the acquisition of gut microbiota was associated with optimal growth trajectories [86]. Additionally, gut microbiota composition differed between SGA rats with and without catch-up growth, suggesting that differences in microbiota may contribute to SGA children's differences in postnatal growth potential [87].

Gut microbiota regulates chondrogenic ossification and the GH-IGF-1 axis in complex ways. By enhancing peripheral tissue sensitivity to GH and raising circulating levels of IGF-1 in mice, the strains of bacterium *Lactobacillus plantarum* promoted the GH axis, overcoming chronic malnutrition-induced GH resistance and developmental delay [88]. Recent research revealed that the cell wall of *Lactobacillus plantarum* enhanced the GH-IGF-1 axis by increasing Nucleotide-binding oligomerization domain-containing protein 2(NOD2) signaling. Malnutrition inhibited the proliferation of small intestinal crypt cells. Activation of NOD2 signaling by *Lactobacillus plantarum* cell wall increases intestinal cell proliferation and improves nutrient absorption, thereby stimulating the activity of the nutrient-sensitive GH/IGF-1/insulin axis and promoting postnatal growth [89]. The length of mice's femurs also grew postnatally, indicating the importance of the gut microbiota in skeletal development. Consistently, Yan et al. demonstrated that the gut microbiota may regulate endochondral ossification by increasing IGF-1 levels through the production of short-chain fatty acids (SCFAs, a byproduct of the gut microbiota), thereby altering longitudinal bone growth [90]. SCFAs could also improve gut barrier function and create an anti-inflammatory environment, and their role in catch-up growth in children with SGA warrants further exploration [90–92].

#### Reprogramming of the hypothalamic–pituitary–adrenal axis

A study of 49 children with IUGR suggested that catch-up growth in children with IUGR may be influenced by intrauterine reprogramming of the HPA axis, and children with increased cortisol secretion may have a higher likelihood of growth failure [93]. Factors including exposure to xenobiotics and psychosocial stress throughout pregnancy, may lead to intrauterine programming and change the hypothalamic–pituitary–adrenal (HPA) axis. Exogenous substances may cause programmed alterations in the fetal HPA axis via epigenetic modifications of essential genes or oxidative stress in the fetal adrenal glands [94]. However, cortisol which is lipophilic and can pass through the placenta, is usually considered the primary mediator. Excess maternal cortisol may continue to impair fetal HPA axis development and result in growth failure [94, 95]. In rats, maternal undernutrition could induce IUGR and overexpose the fetus to maternal corticosterone, leading to increased cortisol secretion in newborns and potential growth retardation [96]. In monkeys, dexamethasone treatment during pregnancy caused a reduction in hippocampal volume and an increase in postnatal plasma cortisol levels. The HPA axis was also enhanced postnatally in fetuses of ewes that were malnourished during the first half of gestation [93, 97]. Because of the hyperactivity of the HPA axis induced by numerous circumstances, elevated cortisol may act by limiting the proteolysis of IGFBP-3, thereby reducing the bioavailability of IGFs [93]. Through what molecular mechanisms intrauterine reprogramming of HPA axis regulates the IGF system to participate in postnatal growth retardation in SGA children will be the focus of future studies.

### Potential therapeutic approaches

Several studies showed that rhGH therapy effectively induced catch-up growth in children with SGA, accompanied by normal body proportions, and improved adult height in the majority of short SGA children [98–100]. However, the growth response to GH was highly variable in all clinical trials involving short children born SGA, and this variability may be attributable, at least in part, to multiple genetic variations [8].

Currently, few studies address the treatment of inadequate catch-up growth among SGA children due to the unknown mechanism behind it. Some animal studies, however, have focused on providing additional nutrients in food restriction models to induce catch-up growth. Based on the similarities in GH resistance and abnormal growth plate development between the food restriction model and the SGA model lacking catch-up growth, these treatments may be applicable to SGA children lacking catch-up growth [101]. Various studies revealed that additional nutritional supplements could lead to catch-up growth in refed animal models by promoting bone growth. In the nutritional restriction and refed rat model, the height of the growth plate fed with casein and whey protein was higher than that provided with a regular diet, and the bone strength and growth rate of rats fed with casein were higher than those fed with whey protein. Higher calcium absorption, induction of IGF-1 secretion, alteration in amino acid profile and digestion velocity may account for this phenomenon [102]. For instance, β Palmitate, the most abundant saturated fatty acid in human milk, was also reported to increase the tibia length and growth plate in refed rats [103]. In addition, a slowly digestible carbohydrate (SDC) diet can improve bone mineral density (BMD), bone mineral content (BMC), growth plate width of limbs, and middle axis bone in a refed rat model [104]. These results suggested that specific dietary patterns and additional nutritional supplements could promote the effects of catch-up growth.

Pharmaceuticals targeting specific protein or gene could be beneficial for SGA children with genetic defects. Recombinant human C-type natriuretic peptide (CNP) analogs are currently authorized in the European Union to treat chondrodysplasia [105]. The fibroblast growth factor receptor three gene (*FGFR3*), a negative bone growth regulator that signals through several different pathways, is the cause of chondrodysplasia. A signaling through the MAPK pathway appears to be the most significant factor in the inhibition of bone growth [106]. CNP is a selective agonist of the natriuretic peptide receptor (NPR2). NPR2 predominantly inhibits MAPK signaling in the growth plate, which speeds up chondrocyte division, matrix production, and cellular hypertrophy [106]. In order to address the decreased chondrogenesis observed in individuals with short stature, Lui et al. created a cartilage-targeted single-chain human antibody fragment (CaAb) designed to deliver therapeutic molecules to the growth plate. In a mouse model with GHD, the subcutaneous injection of the CaAb-IGF-1 fusion protein resulted in an overall increase in growth plate height, while not affecting the proliferation of renal cortical cells, thus minimizing off-target effects on non-cartilage tissues [107]. Given that some short SGA children have similar pathway abnormalities, the therapeutic effect of pharmaceuticals targeting treatments on short SGA children warrants deeper study.

## Conclusions

The mechanisms underlying the absence of catch-up growth in children born with SGA still need to be better understood—several maternal, perinatal and fetal factors affect the ability of SGA infants to catch up. Genetic defects are the most important explanation for the absence of catch-up growth, with signaling pathways in skeletal development, GH resistance, reprogramming of the hypothalamic–pituitary–adrenal axis, epigenetics, and the gut microbiome as possible mechanisms. Ultimately, SGA offspring have impaired postnatal catch-up growth, which increases the risk of dwarfism in adulthood.

Although GH therapy is currently effective in reducing the risk of stunting in most cases of short SGA, the response to GH varies greatly across all clinical trials, and the optimal time to begin GH therapy is debatable due to the uncertain timing of catch-up growth [7–9]. In order to intervene earlier and reduce the likelihood of insufficient catch-up growth, more study on the etiology is required. In terms of other prospective treatment techniques, the idea of translating data from animal trials to clinical application should be pursued.

For decades, there has been little research into the causes and potential mechanisms of catch-up development failure in SGA children. Understanding the etiology and probable causes of catch-up growth failure in SGA children is therefore critical for early detection and treatment options.

## Data Availability

No datasets were generated or analysed during the current study.

## Abbreviations

ALS: Acid-labile subunit
AMP: Adenosine monophosphate
aOR: Adjusted odds ratio
AGA: Appropriate for gestational age
ALSPAC: Avon Parent–Child Longitudinal Study
BMC: Bone mineral content
BMD: Bone mineral density
BMP: Bone Morphogenetic Protein
CaAb: Cartilage-targeted single-chain human antibody fragment
CI: Confidence Interval
CNP: C-type natriuretic peptide
CXXC5: CXXC finger protein 5
CMV: Cytomegalovirus
ECLS-B: Early Childhood Longitudinal Study Birth Cohort
FGF21: Fibroblast growth factor 21
FGFR3: Fibroblast growth factor receptor three gene
FGFs: Fibroblast growth factors
GH: Growth hormone
GHD: Growth hormone deficiency
GHR: Growth hormone receptor
HGF: Hepatocyte growth factor
HSV: Herpes simplex virus
HZ: Hypertrophic zone
HPA: Hypothalamic-pituitary-adrenal
IGF1R: IGF-1 receptors
IGFBP-3: IGF-binding protein 3
IGFBP-5: IGF-binding protein 5
IHH: Indian hedgehog
IGF-1: Insulin-like growth factor 1
IUGR: Intrauterine growth retardation
JAK2: Janus-activated kinase 2
mTOR: Mammalian target of rapamycin
miRNAs: MicroRNAs
MAPK: Mitogen-activated protein kinase
NPR2: Natriuretic peptide receptor 2
NGS: Next-generation sequencing
NAD +: Nicotinamide adenine dinucleotide
NOD2: Nucleotide-binding oligomerization domain-containing protein 2
PTHrP: Parathyroid hormone-related peptide
P/LP: Pathogenic or potentially pathogenic
PZ: Proliferative zone
AKT: Protein kinase B
PI3K: PTHrP Phosphatidylinositol 3-kinase
rhGH: Recombinant human growth hormone
RZ: Resting zone
SHOX: Short stature homeobox
SCFAs: Short-chain fatty acids
STAT5: Signal transducer and activator of the transcription 5
SRS: Silver-Russell syndrome
SIRT1: Sirtuin-1
SDC: Slowly digestible carbohydrate
SGA: Small for gestational age
SDS: Standard deviation scores
TSH: Thyroid stimulating hormone
TGF: Transforming Growth Factor
VZV: Varicella-zoster virus
VLBW: Very low birth weight
